# Differential Risks of the Duration and Degree of Weight Control on Bone Health and Menstruation in Female Athletes

**DOI:** 10.3389/fnut.2022.875802

**Published:** 2022-04-26

**Authors:** Akiko Uchizawa, Emi Kondo, Nemanja Lakicevic, Hiroyuki Sagayama

**Affiliations:** ^1^Graduate School of Comprehensive Human Science, University of Tsukuba, Ibaraki, Japan; ^2^Research Fellow of Japan Society for the Promotion of Science, Tokyo, Japan; ^3^Faculty of Health and Sport Sciences, University of Tsukuba, Ibaraki, Japan; ^4^Sport and Exercise Sciences Research Unit, University of Palermo, Palermo, Italy

**Keywords:** energy availability, female disorder, reproductive, women health, female athlete triad, young athletes, performance, weight cycling

## Introduction

In the 2021 Tokyo Olympics, female athletes reached a record high of 48.8% of all athletes, and women competed in various events. Underneath the success of women in sports, reproductive dysfunction and impaired bone health associated with female relative energy deficiency in sports (RED-S) are considered problematic (1, 2). RED-S refers to “impaired physiological functioning caused by relative energy deficiency and includes, but is not limited to, impairments of metabolic rate, menstrual function, bone health, immunity, protein synthesis and cardiovascular health” and the aetiological factor is low energy availability (EA) (2). Although adverse effects of RED-S on reproductive function and bone health have been consistently reported in female athletes, the magnitude of weight control-associated side effects depends on the type of sport involved in weight control (3, 4). However, menstruation and bone health in female athletes have not been widely discussed and little is known in terms of the duration and degree of weight control and its acute and long-term effects.

## Long-Distance Runners Who Maintain Low Body Weight Over Several Years

Long-distance female runners aim to maintain low body weight or tend to lose weight prior to competition to optimize their time-based performance. Consequently, some long-distance runners experience eating disorders (ED) in the effort to obtain desired low body weights; about half (46%) were classified as “at risk” for developing an ED in a study of collegiate endurance runners from seven US universities (5). In addition, it has been observed that the age of menarche in female runners is 13–15 years, which is later than that in the general population (6, 7). Likewise, female athletes who begin taking part in long-distance competitions before menarche are reported to have menarche at a later age than those who begin after menarche (7). The research on comparing the energy balance is lacking in amenorrheic and eumenorrheic runners, the energy intake of amenorrheic runners seems to be deficient; however, body weight may be maintained due to a decreased resting energy expenditure for maintaining high exercise energy expenditure, thereby not increasing total energy expenditure (8). Regarding bone health, running movements have been associated with both positive and negative effects on the balance of bone remodeling. Physical stimulation of the sole of the foot by running has a positive effect on bone formation, while a negative energy balance due to insufficient energy intake or increased energy expenditure is associated with a decrease in bone collagen formation in young women who exercise regularly (9, 10). World-class middle- and long-distance runners and race walkers were reported to have an EA of 32 kcal/kg fat-free mass (FFM)/day, with about half of them having amenorrhea and 17% of them having a low bone mineral density (BMD) z-score, < −1 (11). Further 51.6% of the female cross-country runners in puberty have been reported to have a low BMD, and 25.8% experienced menstrual irregularity (12).

## Aesthetic Athletes Who Maintain a Low Body Weight From the Early Age

Aesthetic athletes are required to maintain a thin body type and low body weight for an extended period. Aesthetic athletes begin competition at an early age, and maintain low body weight for lengthy periods until they finish competitions. Female figure skating also typically emphasizes slenderness (13), and elite female skaters often begin competing before puberty and practice for more than 30 h/week (13–15). The prevalence of ED is higher among female participants in artistic sports and weight-categorized sports than that in other sports (16). The reported EA in ballet dancers averages 39.5 kcal/kg FFM/day, and 45% of professional ballet dancers reach menarche after 15 years of age, with 65% previously experiencing menstrual dysfunctions (17, 18). The BMD of arms and spine in elite ballet dancers with present and history of amenorrhea was less than elite ballet dancers who had normally menstruating, while the BMD of a leg was comparable (19). Interestingly, the bone density in the lower limbs of figure skaters (mean menarche age: 12.8 years old, range: 11–16 years old) was higher in the landing leg than in the take-off leg (20, 21). It is important to note that ED in athletes increases the risk of injury and can also be a severe problem related to their life due to extreme energy restriction and undernutrition.

## Weight-Categorized Athletes Who Undergo Repeat Rapid Weight Loss in a Short Period

Athletes participating in combat sports that are weight-categorized commonly attempt to gain a body-size advantage by competing in weight divisions that are lower than their natural body weight. To manipulate their weight for the weigh-in ahead of competitions, a combination of chronic and acute weight loss strategies are used (22). To maximize weight loss in the final days leading up to the weigh-in and concurrent competitive events, rapid weight loss (RWL), a weight-manipulation strategy based on drastic food and fluid restriction accompanied by excessive exercise, is frequently utilized by combat sports. Despite a large body of evidence denoting serious acute and chronic health hazards associated with such an approach (23, 24), RWL remains highly prevalent in combat sports, and some athletes repeat RWL up to 10 times per year (25–27). The weight loss range varies; most combat sports athletes lose 2–5% of their body weight in about a week with RWL, while weight loss of 10% or more is not uncommon (26–29). In wrestlers using RWL, the EA during the weight-loss period was 10.7 kcal/kg FFM/day compared to a baseline EA of 43.6 kcal/kg FFM/day calculated from our previous study (30). Of concern, repetitive weight cycling can be especially problematic for female adolescent combat sports athletes. A study on dietary habits in young French female judo athletes revealed that some of the subjects were amenorrheic for 1 year (31). On the other hand, few reports of menstrual disorders or amenorrheic in weight-categorized athletes. Concerning bone health, the BMD of female wrestling and judoka was shown to be 1.21–1.238 g/cm^2^ (32, 33), and the powerful osteogenic stimuli in combat sport provided by their unique biomechanical environment may help prevent bone loss associated with weight loss (33). Nevertheless, have been published just a few reports on BMD in female weight-categorized athletes.

## Discussion

**Figure 1:**
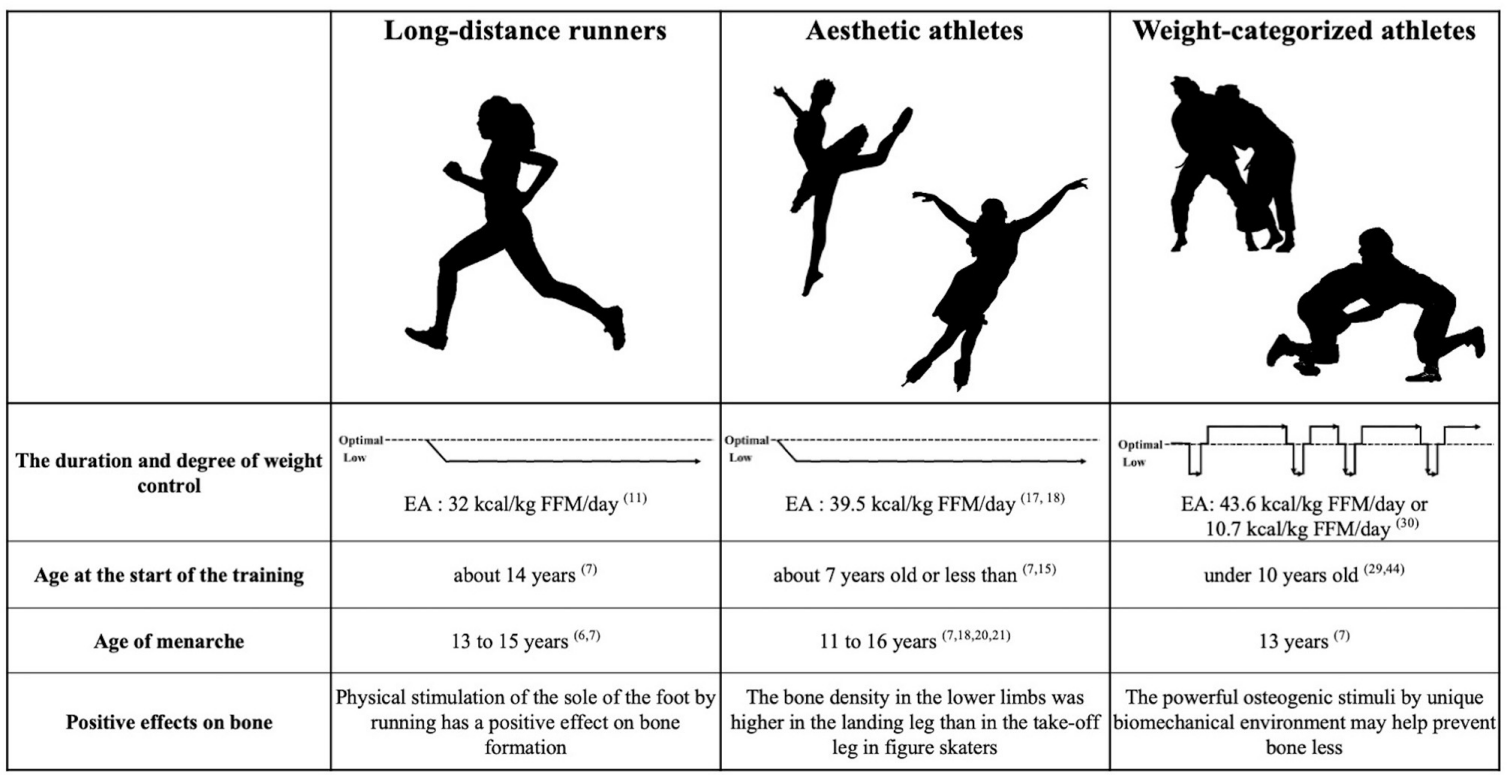
Conceptual diagram of the duration and degree of weight control and the characteristics of each sport. Numbers indicate reference numbers.

It should be noted that EA differs among long-distance runners and weight-categorized sports (Figure 1). Although long-distance runners and aesthetic athletes do not show extremely low EA (<15 kcal/kg FFM/day), continuously low EA (30–45 kcal/kg FFM/day) has been reported over several years of athletic participation (3, 11, 17). In contrast, weight-categorized sports athletes generally showed a daily EA above 45 kcal/kg FFM/day; however, EA can be extremely low during the RWL period (30). Low EA levels cause suppression of estradiol, increased bone resorption, and suppression of bone formation (10). As a result, EA in the RWL period has a negative impact on bone remodeling. However, we previously reported that whole-body BMD in judoka athletes was higher than in endurance athletes, whereas lower limb BMD was equivalent (34). Repetitive whole-body high impact is a feature of combat sports in daily training. Osteogenic stimuli of judo appear to protect athletes from alterations in bone metabolic balance with induced by weight loss cycling (35). The bone remodeling balance is disturbed due to low EA during RWL. However, the totaling period of RWL is 2–3 months per year, and the other approximately 10 months could be spent in recovery period. Thus, the negatively affected bone metabolic status caused by RWL is considered to be improved with subsequent weight regain (33). Intervention studies in amenorrheic athletes suggest that long-term improvement of EA increases luteinizing hormone levels and tends to improve amenorrhea (36). Therefore, obtaining a recovery period for EA may be necessary for the physical development of female athletes. There is a favorable effect on bone health in both long-distance runners and aesthetic athletes; however, in both groups, the positive effect occurs only in the lower limbs, while the BMD of upper limbs appear to be more strongly affected by prolonged low energy intake than lower limbs (20, 21, 34). Whereas in many sporting activities, bone gains only in the areas of impact, in combat sports such as judo and wrestling constantly produce strains that are distributed throughout the entire skeleton (33).

The menarche age is generally around 12 years (37), and if no menstruation occurs after 15 years of age, primary amenorrhea is suspected. The delay of menarche is a significant adverse factor for bone formation during puberty, as bone mineral content reaches 90% of the bone peak by the age of 18 years, with 25% of the bone peak formed during the 2 years before and after menarche (38, 39). Indeed, there is a positive correlation between the later age of menarche and BMD (40). The following menarche ages were reported in Polish female athletes: 14.2 years in long-distance runners, 14.1 years in marathon runners, and 13.0 years in Judo athletes (7). In another study, the low BMD group (<-2.0 z-score) had a menarche age of 13.29 years, which was significantly more delayed than that of the comparable BMD group (-1.0 to−2.0 z-score), 12.27 years (41). The lower whole-body BMD in long-distance runners and aesthetic athletes may be due to delayed menarche age, despite the positive effects of athletic characteristics on BMD formation. Between the age ranges of 13–14 and 17–18 years, a significant increase in the BMD of non-runners was reported; however, no increase in the BMD of runners was observed (42). The EA for optimal physical function is generally over 45 kcal/kg FFM/day; however, a much higher EA may be necessary for pubertal patients individuals, who are still in growth and development phase. Runners and aesthetic athletes have been reported to have chronic energy deficiency following the start of their sports (12, 17), and most aesthetic athletes begin their competitions before menarche. On average, long-distance runners begin competition in secondary school, and those who began competing before menarche had a later age of menarche (7). Thus, long-distance runners and aesthetic athletes are considered to have insufficient growth during puberty and delayed menarche age due to chronic energy deficiency. Thus, significant attention should be paid to the delay in menarche during puberty in female athletes.

Prolonged energy deficiency over several years, especially during the puberty period, may lead to insufficient growth in female athletes (2). Although female runners and aesthetic athletes do not have experience extreme energy restriction associated with RWL, they may continue to experience have long-term adverse effects on menstruation and bone health. Weight-categorized female athletes may be seemingly less likely to develop menstrual disorders and low BMD, but dietary restrictions associated with RWL may induce ED in adolescent female athletes (43). Therefore, although RWL practices in juniors may not directly affect low BMD, the development of ED and anorexia nervosa due to repeated RWL may lead to chronic low EA; thus, there may be some delay in menarche or physical growth during puberty caused by RWL. In addition, fluid restriction, one of the principal methods of RWL, increases the risk of heatstroke, especially in adolescents (44). Taken together, as we recently reported (27), and RWL is not recommended for female athletes and a strategy for weight control that considers the menstrual cycle, and the resulting effects of water fluctuation is required. To date, little data are available on the effect RWL has on menstruation, and further research is required to elucidate this relationship. Athletes who take part in weight-categorized sports and engage in weight control practices are most influenced by their coaches, with few to no athletes seeking advice from staff with professional expertise (e.g., dietitians or doctors) (27). The normal development of reproductive functions around puberty, particularly for females, has a significant impact on their future life (45); thus, a collaboration with qualified staff is required. Notably, health consequences related to repeated cycles of acute and chronic weight cycling may prevent many athletes from pursuing longer athletic careers, and this is an issue that should not be overlooked.

As stated, risks to menstruation and bone health differ based on the duration and degree of weight control, and it is necessary to provide nutritional and training advice that is tailored to various athletes' characteristics. Long-distance runners and aesthetic athletes particularly require nutritional and training strategies to achieve adequate growth during puberty. Future studies should be conducted, especially in pre-pubertal athletes, to further explore these relationships.

## Author Contributions

AU, EK, and HS contributed to conception and design of the study and firstly drafted the manuscript. AU, EK, NL, and HS edited and revised and approved the final version of the manuscript. All authors contributed to the article and approved the submitted version.

## Conflict of Interest

The authors declare that the research was conducted in the absence of any commercial or financial relationships that could be construed as a potential conflict of interest.

## Publisher's Note

All claims expressed in this article are solely those of the authors and do not necessarily represent those of their affiliated organizations, or those of the publisher, the editors and the reviewers. Any product that may be evaluated in this article, or claim that may be made by its manufacturer, is not guaranteed or endorsed by the publisher.
